# Development and interpretation of a pathomics-based model for the prediction of microsatellite instability in Colorectal Cancer

**DOI:** 10.7150/thno.49864

**Published:** 2020-09-02

**Authors:** Rui Cao, Fan Yang, Si-Cong Ma, Li Liu, Yu Zhao, Yan Li, De-Hua Wu, Tongxin Wang, Wei-Jia Lu, Wei-Jing Cai, Hong-Bo Zhu, Xue-Jun Guo, Yu-Wen Lu, Jun-Jie Kuang, Wen-Jing Huan, Wei-Min Tang, Kun Huang, Junzhou Huang, Jianhua Yao, Zhong-Yi Dong

**Affiliations:** 1 Information Management and Big Data Center, Nanfang Hospital, Southern Medical University, Guangzhou, China.; 2 AI Lab, Tencent, Shenzhen, China.; 3 Department of Radiation Oncology, Nanfang Hospital, Southern Medical University, Guangzhou, China.; 4 Department of Pathology, Union Hospital, Tongji Medical College, Huazhong University of Science and Technology, Wuhan, Hubei, China.; 5 Indiana University Bloomington, Bloomington, USA.; 6 Tongshu Biotechnology Co., Ltd. Shanghai, China.; 7 Tencent Healthcare, Shenzhen, China.; 8 Department of Medicine, Indiana University School of Medicine, Indianapolis, IN, USA.; 9 Regenstrief Institute, Indianapolis, IN, USA.; 10 Department of Computer Science, Technical University of Munich, Munich, Germany.

**Keywords:** microsatellite instability, colorectal cancer, pathomics, multi-omics, ensembled patch likelihood aggregation (EPLA)

## Abstract

Microsatellite instability (MSI) has been approved as a pan-cancer biomarker for immune checkpoint blockade (ICB) therapy. However, current MSI identification methods are not available for all patients. We proposed an ensemble multiple instance deep learning model to predict microsatellite status based on histopathology images, and interpreted the pathomics-based model with multi-omics correlation.

**Methods:** Two cohorts of patients were collected, including 429 from The Cancer Genome Atlas (TCGA-COAD) and 785 from an Asian colorectal cancer (CRC) cohort (Asian-CRC). We established the pathomics model, named Ensembled Patch Likelihood Aggregation (EPLA), based on two consecutive stages: patch-level prediction and WSI-level prediction. The initial model was developed and validated in TCGA-COAD, and then generalized in Asian-CRC through transfer learning. The pathological signatures extracted from the model were analyzed with genomic and transcriptomic profiles for model interpretation.

**Results:** The EPLA model achieved an area-under-the-curve (AUC) of 0.8848 (95% CI: 0.8185-0.9512) in the TCGA-COAD test set and an AUC of 0.8504 (95% CI: 0.7591-0.9323) in the external validation set Asian-CRC after transfer learning. Notably, EPLA captured the relationship between pathological phenotype of poor differentiation and MSI (*P* < 0.001). Furthermore, the five pathological imaging signatures identified from the EPLA model were associated with mutation burden and DNA damage repair related genotype in the genomic profiles, and antitumor immunity activated pathway in the transcriptomic profiles.

**Conclusions:** Our pathomics-based deep learning model can effectively predict MSI from histopathology images and is transferable to a new patient cohort. The interpretability of our model by association with pathological, genomic and transcriptomic phenotypes lays the foundation for prospective clinical trials of the application of this artificial intelligence (AI) platform in ICB therapy.

## Introduction

Microsatellite instability (MSI) is a hypermutator phenotype that occurs in tumors with DNA mismatch repair deficiency (dMMR) 1, which is reported as a hallmark of hereditary Lynch syndrome (LS)-associated cancers 2 and observed in about 15% of colorectal cancer (CRC) 3. MSI has been identified as a favorable prognostic factor but a negative predictor for adjuvant chemotherapy in stage II CRC 4. More importantly, recent studies have demonstrated MSI or dMMR is correlated to an increased neoantigen burden that sensitizes the tumor to immune checkpoint blockade (ICB) treatment 5. Further investigations have suggested that the benefit of ICB treatment for patients with MSI is not limited to specific tumor types but to all solid tumors 6, which established the crucial role of MSI in predicting the efficacy of immunotherapy for advanced solid tumors, especially CRC.

MSI or dMMR testing has traditionally been performed in patients with CRC and endometrial cancer to screen for LS-associated cancer predisposition 7. Recently, with the U.S. Food and Drug Administration (FDA) designation of MSI/dMMR as a favorable predictor of anti-programmed death-1 (PD-1) therapy 8, the clinical demand for MSI/dMMR testing has increased dramatically. However, in clinical practice, not every patient is tested for MSI, especially in those cancers with lower occurrences of MSI or in patients in developing countries, because it requires additional genetic or immunohistochemical tests which are costly and time-consuming. Additionally, various existing MSI testing methods show different sensitivities and specificities, leading to the disunity of results 9, 10. Therefore, there are both opportunities and challenges that lie ahead in developing an MSI testing method that is available for all cancer patients.

The emergence of computational pathology have provided an opportunity for the detection of MSI because pathology slides are produced for almost every patient diagnosed with cancer; these slides can be digitized into whole slide images (WSIs) 11. WSI not only reveals the tissue spatial arrangement of tumor cells at low magnification, but also the cell structure at high magnification 12. Furthermore, histopathology images also show the immunologic microenvironment of tumors 13. The cell level phenotypes presented in WSI are affected by genotypes such as MSI at the molecular scale. With the continuous penetration of artificial intelligence (AI) into the field of medical imaging, researchers have sought solutions based on deep learning, a research area in AI, in a wide range of medical problems, such as prediction of gene mutations 14 and tumor-infiltrating lymphocytes 12, and cancer screening 15, 16. Whereas traditional machine learning depends largely on human-selected features 17, deep learning can learn features from the data, which makes it possible for researchers to discover untapped information 18, 19. Previous studies have suggested that deep learning can discover regions that contribute to microsatellite (MS) status with special pathomorphological characteristics 20, but the applicability of the model in the Asian population remains in question because of the great variation in demographics and data preparation. The inability to interpret the extracted signatures and the predictions made by the model is considered to be one of the major issues that limit the acceptance of AI models in medicine 21.

In this study, we developed a multiple-instance-learning (MIL)-based deep learning model to predict MS status from histopathology images. The model, for which we proposed as Ensemble Patch Likelihood Aggregation (EPLA), combined both deep learning and traditional machine learning techniques. It was trained using the TCGA-COAD data set, and then transfer learning was implemented to fine tune the model using an Asian-CRC cohort curated locally, which enhanced the generalizability of this model. More importantly, we also demonstrated the interpretability of the model by identifying the crucial pathological signatures generated by the MIL model and linking them with MSI genomic and transcriptomic profiles.

## Materials and Methods

### Patient cohorts and dataset partition

In this study, whole slide images (WSIs) of two large cohorts were collected, and an MS label was assigned to each WSI based on the patient's microsatellite measurement. The first cohort (TCGA-COAD), retrieved from The Cancer Genome Atlas, comprised 429 frozen tissue slides diagnosed as colon adenocarcinoma (COAD) with stage I to IV. MSI score of each sample within the cohort was measured using the MSIsensor algorithm based on tumor-normal paired genome sequencing data 22; tumors with MSIsensor scores of ≥ 10 were defined as MSI, whereas those with MSIsensor scores of < 10 were defined as microsatellite stability (MSS) 23. In this cohort, 358 cases were labeled as MSS and 71 cases were labeled as MSI. The second cohort (Asian-CRC), collected from Tongshu Biotechnology Co., Ltd, consisted of 785 formalin-fixed paraffin-embedded (FFPE) sections diagnosed with CRC of all stages, which were provided from three medical centers in China. Patients in the Asian-CRC group were analyzed by an MSI detection kit (Shanghai Tongshu Biotechnology Co., Ltd.) that detects five microsatellite loci (BAT-25, BAT-26, D5S346, D2S123 and D17S250) based on multiplex PCR-capillary electrophoresis 24; tumors with instability in ≥ 2 out of five microsatellite loci were classified into the MSI-high (MSI-H) group, and the rest were assigned into the MSI-low (MSI-L)/MSS group, following the recommendations and guidelines on MSI testing for CRC 24, 25. Thus, 164 cases were identified as MSI-H, and 621 cases were identified as MSI-L/MSS. The details of the two cohorts are summarized in Table S1. This study was approved by the Institutional Ethical Review Boards of Nanfang Hospital (NFEC-2020-055), and patient consents were obtained.

The TCGA-COAD cohort was split into separate training and test sets at a 7:3 ratio using stratified sampling, in order to maintain the same ratio of positive to negative samples in the training set and test set. The training set was used for hyper-parameter tuning based on cross-validation, whereas the test set was used for the evaluation of generalization performance, and the independent Asian-CRC cohort for external validation.

### ROI delineation, tiling, and data preprocessing

All WSIs were digitalized at 20× objective lens with a predefined pixel resolution (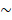
0.5μm/pixel). In order to reduce the influence of unrelated areas and alleviate the workload of the classification method, regions of carcinoma (ROIs) on WSIs were manually annotated by expert pathologists, according to the following rules: (1) the tumor cells should occupy more than 80% of a ROI, i.e., the interstitial component is less than 20%; and (2), obvious interfering factors, including creases, bleeding, necrosis and blurred areas, should be excluded. The annotation was performed using Aperio ImageScope (Aperio Technologies, Inc.).

Given the extremely large image size (typically 100,000 × 50,000 pixels) of a WSI, the WSIs were subsequently tiled into 512×512 patches. Only patches having a greater than 80% overlap with the carcinoma ROI were used for the following analysis. The number of patches per WSI in TCGA-COAD ranges from 22 to 2357 (average 224), whereas the Asian-CRC ranges from 5 to 3718 (average 338) (Table S1).

Data augmentation and normalization were applied for training patches, whereas only normalization was employed for test patches. Data augmentations used in our work included random horizontal flipping and random affine transformation of the patches (keeping the center invariant). Finally, the augmented patches were center cropped to 224 pixels × 224 pixels similar to Campanella's study 26, following a z-score normalization on RGB channels.

### Multiple Instance Learning (MIL)-based deep learning pipeline

Our MIL-based deep learning pipeline presented two predictions: patch-level and WSI-level. Due to the large image size and heterogeneity in tumors, the WSI was first divided into small patches, and then the patch likelihoods were aggregated in an ensemble classifier to obtain the WSI-level prediction. Therefore, our method was termed Ensemble Patch Likelihood Aggregation (EPLA).

During the patch-level prediction, a residual convolutional neural network (ResNet-18) was trained to compute the patch likelihood in a MIL paradigm where the patches were assigned with the WSI's label. Binary cross-entropy (BCE) loss was utilized to optimize the network using a mini-batch gradient descent method.

We developed two independent MIL methods to aggregate the patch likelihoods: Patch Likelihood Histogram (PALHI) pipeline and Bag of Words (BoW) pipeline, which were inspired by the histogram-based method and the vocabulary-based method, respectively. In PALHI, a histogram of the occurrence of the patch likelihood was applied to represent the WSI, whereas in BoW, each patch was mapped to a TF-IDF floating-point variable, and a TF-IDF feature vector was computed to represent the WSI. Traditional machine learning classifiers were then further trained using these feature vectors to predict the MS status for each WSI. Here, Extreme Gradient Boosting (xgboost), a kind of gradient boosted decision tree, was employed in the PALHI pipeline. Naïve Bayes (NB) was used in the BoW pipeline. During the training of the WSI-level classifier, the hyperparameters were determined based on the cross-validation on the training set, using WSI-level ROCAUC as the performance metric. During WSI-level prediction, the results of PALHI and BoW classifiers were then ensembled to obtain the final prediction 27.

The initial parameters of the model were trained in the training set of TCGA-COAD, and the transfer learning technique was implemented using the Asian-CRC data to generalize the model across cohorts with a high degree of heterogeneity. The transfer learning was conducted by reusing the model weights in the patch-level discriminators and then fine-tuning the weights using a small amount of labeled Asian-CRC data. In addition, we gradually added more Asian-CRC data for model fine-tuning to explore the impact on model performance. All codes were implemented in Python 3.6.5 and run on a workstation with Nvidia GPUs (P40). As for the minimal requirement, a desktop with CPUs and the above dependencies can run our algorithm for inference, which is widely available and easy-to-use for physicians and biologists. The average time for the completion of a single patient test is 0.5118s on a P40 workstation and 20.9291s on a regular CPU machine (i5-9500, 3.00GHz, 16GB).

### Multi-omics correlation analysis of pathological signatures

#### Identification of pathological signatures of importance

The occurrence histograms in the PALHI and the TF-IDF feature vector in BoW were the pathological signatures generated by our model. The importance of each signature was measured by its contribution weight to the final WSI-level prediction for discovering top pathological signatures. The top pathological signatures were evaluated by Wilcoxon Rank Sum tests for significance and then sent for genomic and transcriptomic correlation analysis.

#### Genomic correlation analysis

The DNA mutation profile of TCGA-COAD was retrieved from cBioPortal 28. The synonymous mutations were excluded from the following correlation analysis. For a particular gene set, as long as there was a non-synonymous mutation in any of its gene members, it would be defined as deficient.

The relationship between MSI and some mutation indexes has been reported in previous literature, including INDEL and tumor mutation burden (TMB) 29. INDEL mutations refer to a variant type caused by sequence insertion (INS) or deletion (DEL) and can be calculated as the frequency of DEL and INS mutations. As the mutation data was profiled by the whole exome sequencing, TMB is defined and calculated as the total number of somatic nonsynonymous mutations divided by size of the exonic region of the entire genome 30. To explore the relationship between the pathological signatures and these known genomic biomarkers, they were first normalized to a range of 0 to 1 and then visualized in a heat map using the R package *pheatmap*, during which unsupervised clustering was applied using Ward's minimum variance method.

#### Transcriptomic correlation analysis

The mRNA expression profile of TCGA-COAD, retrieved from cBioPortal, was normalized using the RSEM method 31. Gene co-expression network analysis (WGCNA) is a bioinformatics method based on expression data and is typically used to identify gene modules with highly synergistic changes 32. We first constructed a gene co-expression network for the mRNA expression profile using the R package *WGCNA*, during which the soft threshold for the network was set to the recommended value selected by the function *pickSoftThreshold* (Figure S1). Setting the minimum module size to 100 and other parameters to default, we identified 24 transcriptomic modules (Figure S2). The biological functions of the modules were annotated by the Gene Ontology (GO) over-representation test using the R package *clusterProfiler* 33, during which the Benjamini-Hochberg method was used to adjust *P* value for controlling false discover rate. Only those GO terms with adjusted *P* values lower than 0.05 were considered significantly enriched in a particular module. After that, we calculated Spearman's rank correlation coefficients for each pair of modules and pathological signatures to recognize the modules of interest.

An immune cytolytic activity (CYT) score, defined as the geometric mean of transcript levels of *GZMA* and *PRF1* 34, as well as a CD8^+^ T-effector gene set (*CD8A*, *IFNG*, *GZMA*, *PRF1*, *CXCL9*, *CXCL10*, *TBX21*, *GZMB*) 35 was quantified from the RNA-seq data, and subsequently associated with pathological signatures to characterize the correlation with anti-tumor immunity.

### Statistical analysis

The ROC curves were drawn using *pROC* and *ggplot2* in R (version 3.6.1). The area under the ROC curve and confidence intervals were calculated in *pROC*. The significance of AUC differences was tested using the Wald test statistic 36. The optimal cutoff points of the ROC curves were estimated using the Youden Index 37. The Wilcoxon Rank Sum test was used to compare two paired groups and visualized as a boxplot using R package *ggpubr*. Spearman's rank correlation coefficients were used for correlation analysis.

## Results

### Development and performance evaluation of EPLA model

**Figure 1:**
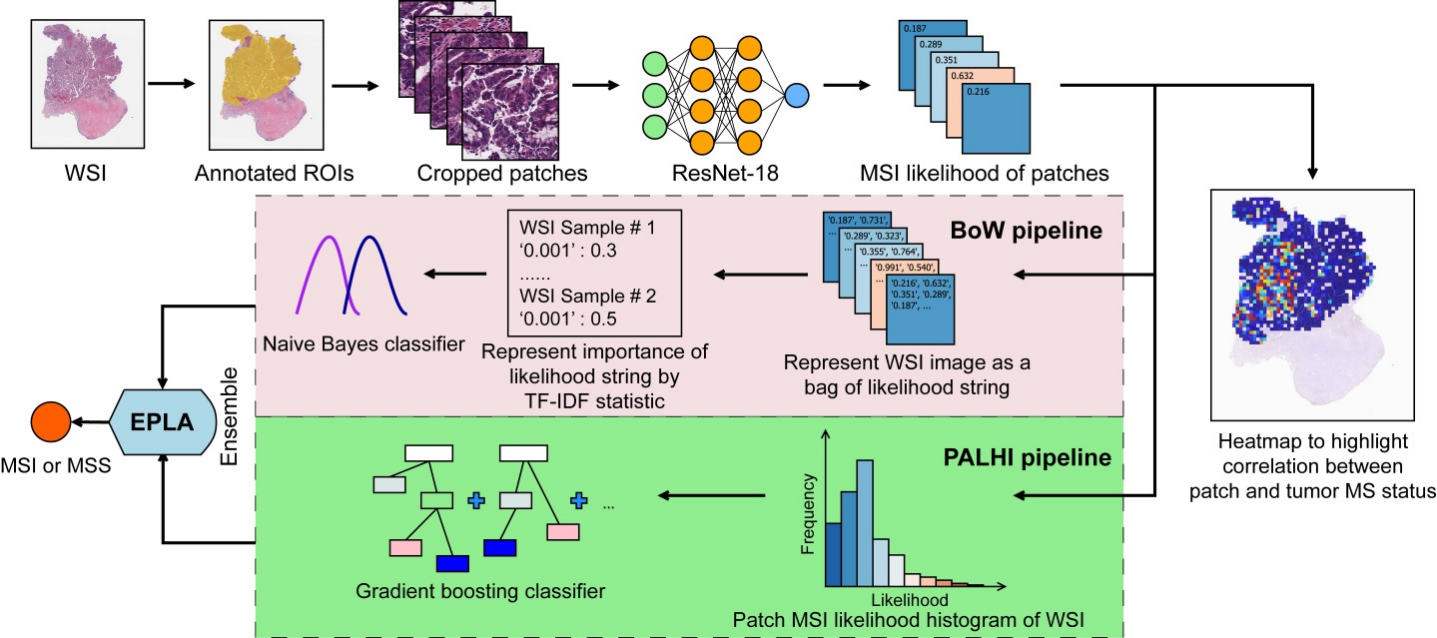
** Overview of the Ensemble Patch Likelihood Aggregation (EPLA) model.** A whole slide image (WSI) of each patient was obtained and annotated to highlight the regions of carcinoma (ROIs). Then, patches were tiled from ROIs, and the MSI likelihood of each patch was predicted by ResNet-18, during which a heat map was shown to visualize the patch-level prediction. Then, PALHI and BoW pipelines integrated the multiple patch-level MSI likelihoods into a WSI-level MSI prediction, respectively. Finally, ensemble learning combined the results of the two pipelines and made the final prediction of the MS status.

The pathomics-based model named EPLA was developed in the training set of the TCGA-COAD cohort (7:3 for training and test), which consisted of two consecutive stages: patch-level prediction and WSI-level prediction (Figure 1). Briefly, a WSI was annotated to delineate the region of carcinoma (ROI). The ROI was tiled into patches, which were subsequently fed to a residual convolutional neural network (ResNet-18) to obtain the patch-level MSI prediction. Then, we trained two independent MIL pipelines to integrate multiple patch-level predictions into an MSI score at the WSI level: the PAtch Likelihood HIstogram (PALHI) pipeline and the Bag of Words (BoW) pipeline. To obtain the optimal convex combination of the two MIL methods, we employed ensemble learning to eventually obtain the predicted MS status of the patient (Figure 1).

**Figure 2:**
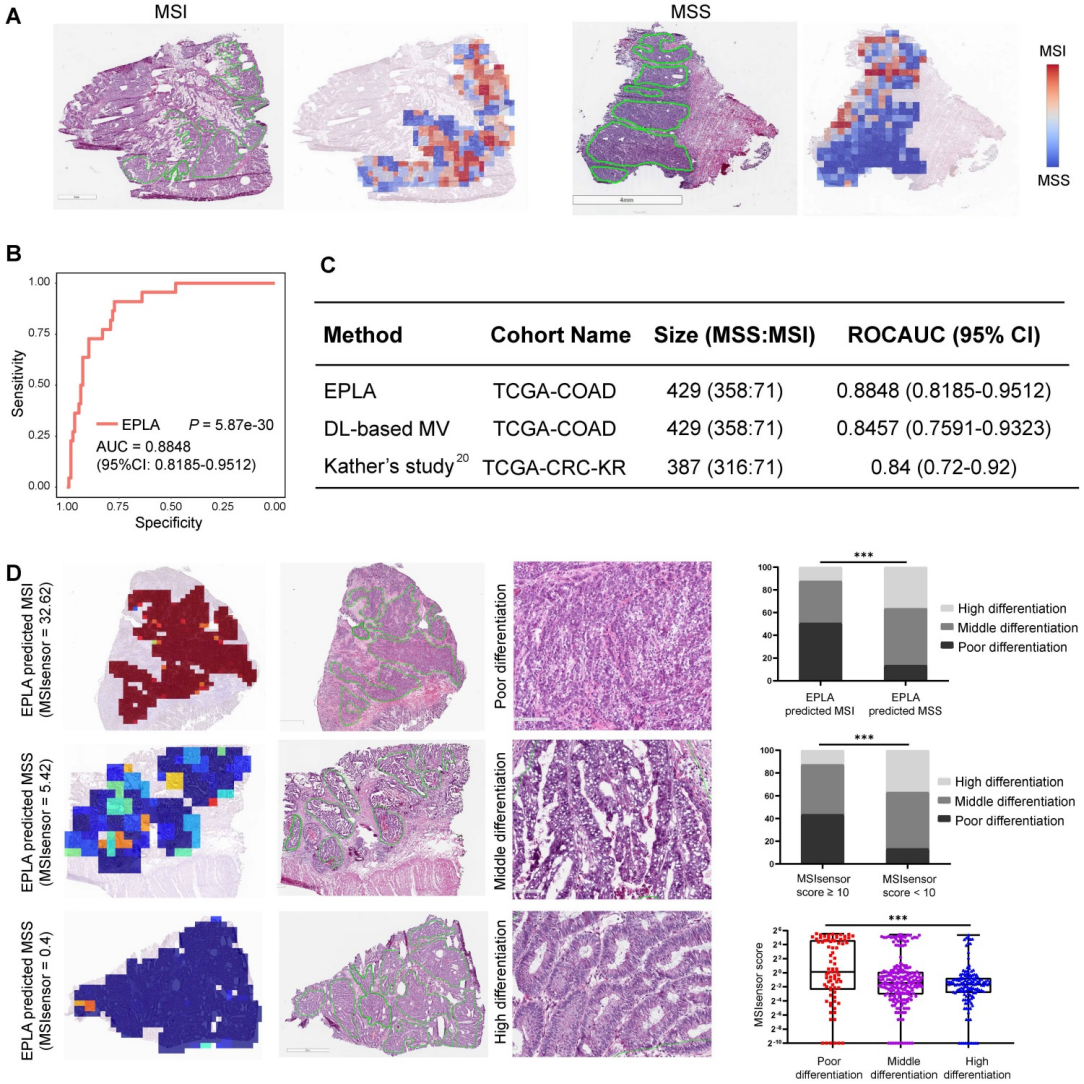
** Validation of the EPLA and comparison with DL-based MV in the TCGA cohort.** (**A**) Representative heat maps of MSI and MSS cases at the patch-level prediction stage. Color bars show the MSI likelihood of each patch. (**B**) Receiver operating characteristic (ROC) curve of EPLA. The *P* value was calculated by the Wald test. (**C**) Summary of EPLA and DL-based MV. DL-based MV was re-implemented from a voting-based model in Ref.20. The last line of the table summarizes the performance of the original DL-based MV model. (**D**) Correlation of the degree of differentiation with EPLA-predicted MS status and MSIsensor score. DL-based MV, deep-learning based majority voting; EPLA, Ensemble Patch Likelihood Aggregation. Significance values: *** *P* < 0.001.

The performance of the EPLA model was measured in the TCGA-COAD test set. Two representative heat maps providing the patch level prediction, for an MSI case and an MSS case respectively, are shown in Figure 2A. The EPLA model achieved an AUC of 0.8848 (95% CI: 0.8185-0.9512) at the WSI level (Figure 2B) and outperformed the state-of-the-art Deep-Learning based Majority Voting method (denoted as DL-based MV) in Kather's study 20, which trained a ResNet for patch-level predictions and then took the majority of these predictions as the final MS status of the patient (Figure 2C). To directly compare our method with the DL-based MV in the same test set, we implemented DL-based MV method in the TCGA-COAD cohort, and achieved an AUC of 0.8457 (95% CI: 0.7591-0.9323) consistent with the result in Kather's study (Figure 2C).

We further compared the specificity and sensitivity of the two components of the EPLA (i.e., PALHI and BoW) to that of DL-based MV (Table S2). We found that BoW achieved higher specificity (89.5% vs 75.2%) and PALHI was superior in terms of sensitivity (86.4% vs 81.8%). The ensembled EPLA classifier combined the advantage of its two components and thus obtained both superior specificity and sensitivity compared to the DL-based MV (Table S2). Representative heat maps of the discrepant cases are shown in Figure S3. These cases were correctly predicted by EPLA but mistakenly classified by DL-based MV.

Additionally, an exploratory analysis was undertaken to identify the pathological phenotype recognized by EPLA. Of note, EPLA captured the relationship between the degree of differentiation (poor, middle or high differentiation) and MS status. Tumors with higher MSIsensor score or were predicted as MSI by EPLA model showed high proportion of poor differentiation, while lower MSIsensor score or predicted MSS tumors were demonstrated increasing proportion of high and middle differentiation (*P* < 0.001), which supports the inner relationship between EPLA model and pathological morphology (Figure 2D).

### External validation of EPLA in an Asian-CRC cohort

**Figure 3:**
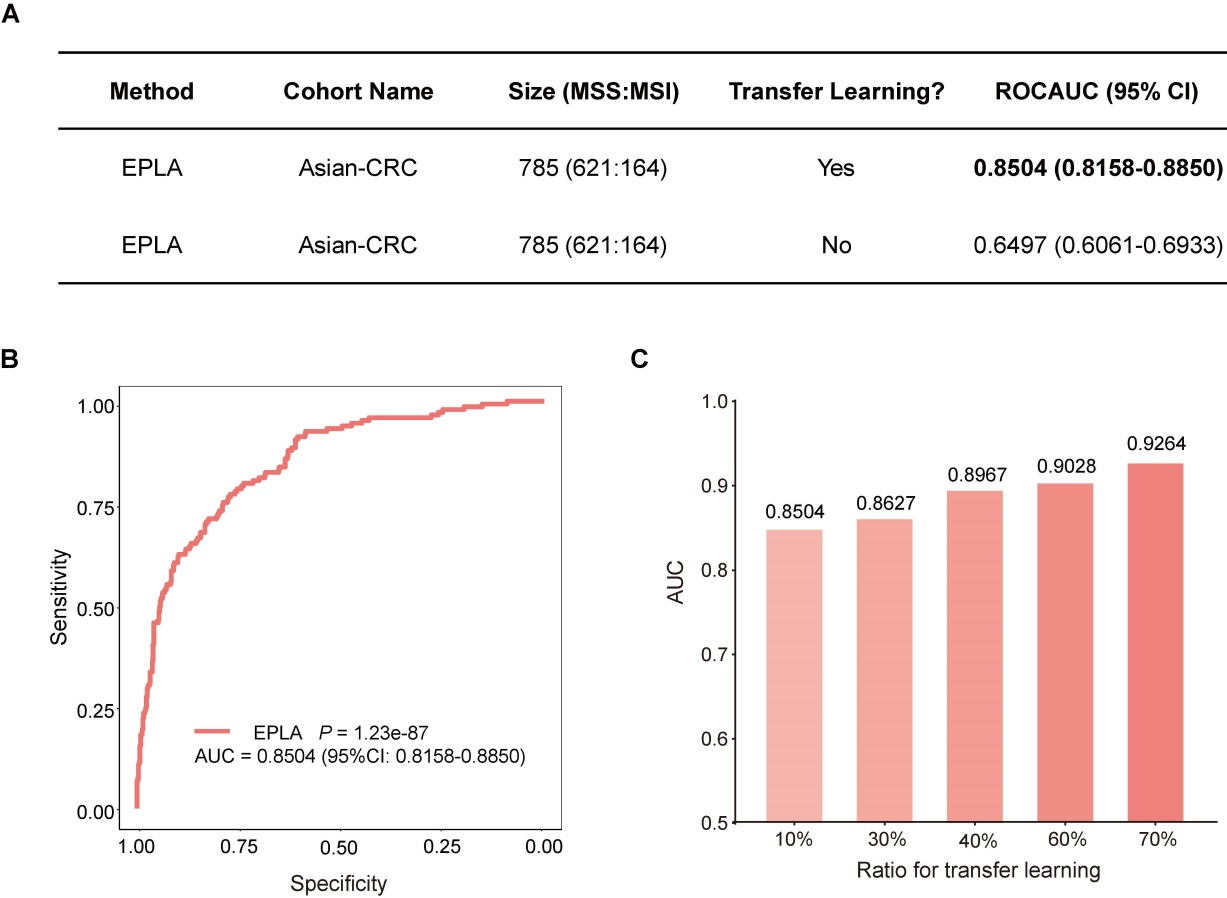
** Generalization performance of the EPLA in an Asian cohort.** (**A**) Summary of the performance of EPLA in Asian-CRC with or without transfer learning. When using transfer learning, 10% of cases from Asian-CRC were used for model fine-tuning. (**B**) The Receiver operating characteristic (ROC) curve of EPLA in the Asian-CRC after transfer learning. (**C**) ROCAUCs of the model in Asian-CRC with increasing proportions of cases for transfer learning. EPLA, Ensemble Patch Likelihood Aggregation; CRC, colorectal cancer.

We further measured the generalizability of our model in an Asian-CRC cohort. It was noteworthy that there existed great differences between the Asian-CRC cohort and the TCGA-COAD cohort, not only in patient race but also in the slide preparation techniques (Table S1). As a consequence, the EPLA model trained on TCGA-COAD only achieved an AUC of 0.6497 (95% CI: 0.6061-0.6933) on the external validation data set Asian-CRC (Figure 3A). Considering the wide variations in medical practice, we therefore applied transfer learning to generalize the EPLA model by fine-tuning our model using only 10% of cases from Asian-CRC, and thus achieved an AUC of 0.8504 (95% CI: 0.8158-0.885) in the remaining data set (Figure 3A-B). Moreover, we analyzed the performance of the EPLA model for MS status prediction across tumor stages; the EPLA model achieved high prediction performance in both non-metastatic and metastatic CRC cases, with an AUC of 0.8768 (95% CI: 0.8427-0.9110) in the stage I-III subgroup and an AUC of 0.8242 (95% CI: 0.7460-0.9023) in the stage IV subgroup, indicating the robustness of the model in predicting MS status of CRC (Figure S4).

We subsequently evaluated the amount of data needed for transfer learning by increasing the proportion of cases from Asian-CRC for model fine tuning. The performance of the fine-tuned model steadily improved, resulting in 0.8627 (95% CI: 0.8208-0.9045), 0.8967 (95% CI: 0.8596-0.9338), 0.9028 (95% CI: 0.8534-0.9522) and 0.9264 (95% CI: 0.8806-0.9722) AUCs in the ratios of 30%, 40%, 60% and 70%, respectively, implying that transfer learning was an effective measure to overcome the heterogeneity between different cohorts (Figure 3C).

### Identification of top pathological signatures from the EPLA model

**Figure 4:**
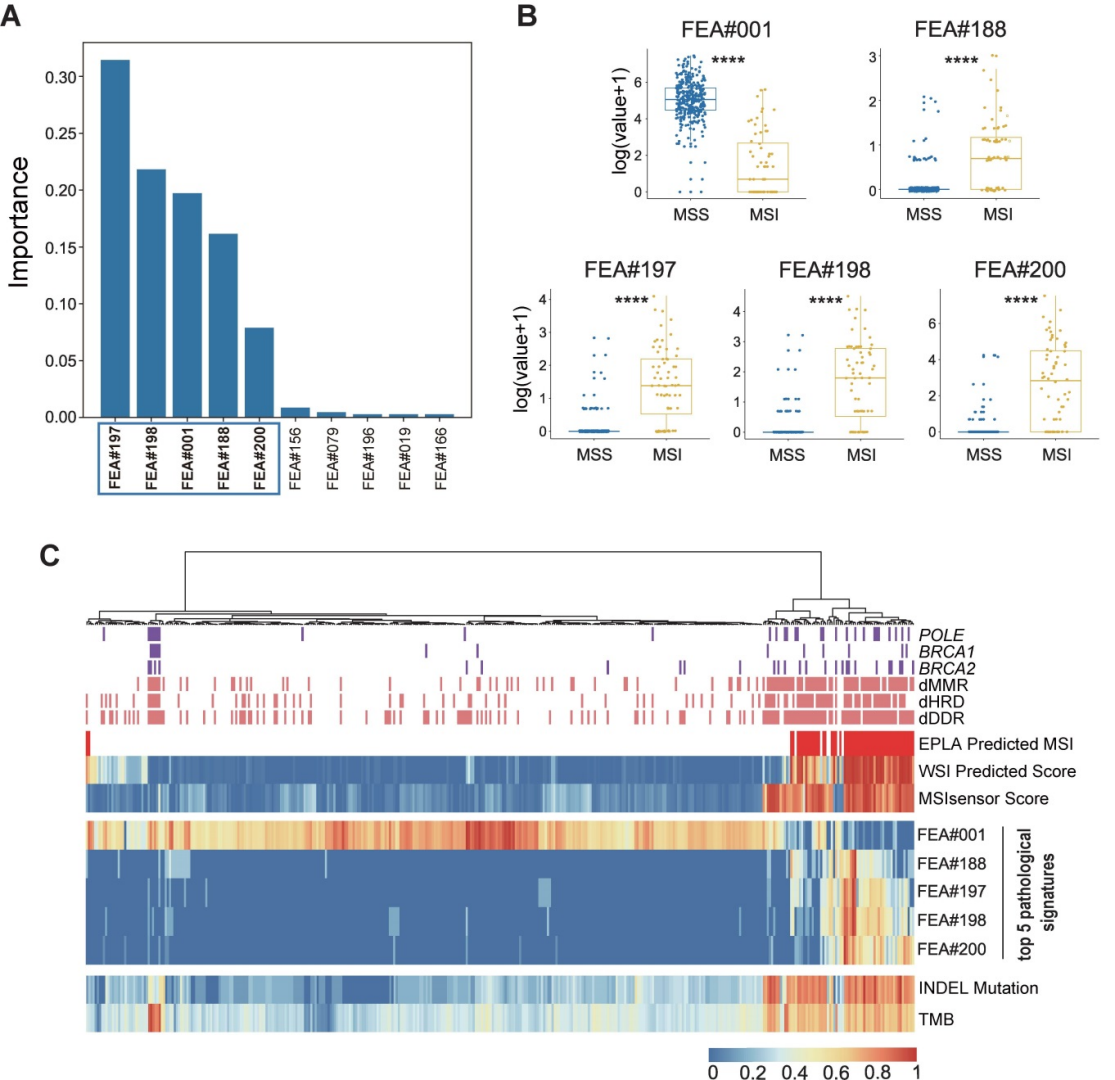
** Identification and genomic correlation analysis of top pathological signatures.** (**A**) Importance ranking of the top ten pathological signatures extracted from EPLA. (**B**) Boxplots of the five pathological signatures between MSI and MSS groups. Significance values: **** *P* < 0.0001. (**C**) Heat map with unsupervised clustering showing the correlation between genomic landscape and top pathological signatures in each patient. Each column corresponds to a patient in the TCGA-COAD cohort. All continuous variables are normalized to a range of 0 to 1. EPLA, Ensemble Patch Likelihood Aggregation; FEA, feature; INDEL: insertion-deletion, TMB: tumor mutation burden, MMR: mismatch repair, DDR: DNA damage response and repair, and HRD: homologous recombination deficiency.

To gain insight into the MSI prediction mechanism of the model, we explored the contribution of the pathological signatures extracted from the EPLA model to the prediction of MSI in TCGA-COAD. The ranking of significance of the top ten pathological signatures is shown in Figure 4A. Given that the top five pathological signatures (FEA#197, FEA#198, FEA#001, FEA#188 and FEA#200) were significantly more important than the others, they were selected for subsequent analysis. Among them, FEA#001 had a significantly higher value (*P* < 0.0001) for patients in the MSS group, while the other four (FEA#188/197/198/200) had significantly higher values (*P* < 0.0001) in the MSI group (Figure 4B). Then we employed molecular-level association analysis to link the pathological signatures and the genetic alterations, which enhanced the clinical interpretation and application value of our AI method.

### Association of the EPLA related pathological signatures and genomic landscape

Cluster analysis in Figure 4C shows that patients with a high value of FEA#001 were mainly MSS with normal function in DNA repair-related pathways consisting of mismatch repair (MMR), DNA damage response and repair (DDR), and homologous recombination deficiency (HRD). On the contrary, patients with high levels of FEA#188/197/198/200 were mainly due to MSI with deficient DNA repair related pathways, namely deficient-MMR (dMMR), deficient-DDR (dDDR) and deficient-HRD (dHRD). In addition, mutations of several representative genes in these pathways, including *POLE*, *BRCA1*, and *BRCA2*, also demonstrated a consistent finding. Moreover, since recent evidence suggested MSI was significantly related to TMB, especially INDEL mutation load 29, we assessed the relation between the pathological signatures and these known biomarkers and found that high TMB and INDEL mutation load were often accompanied by low FEA#001 and high FEA#188/197/198/200 (Figure 4C).

### Association of the EPLA related pathological signatures and transcriptomic pathway

**Figure 5:**
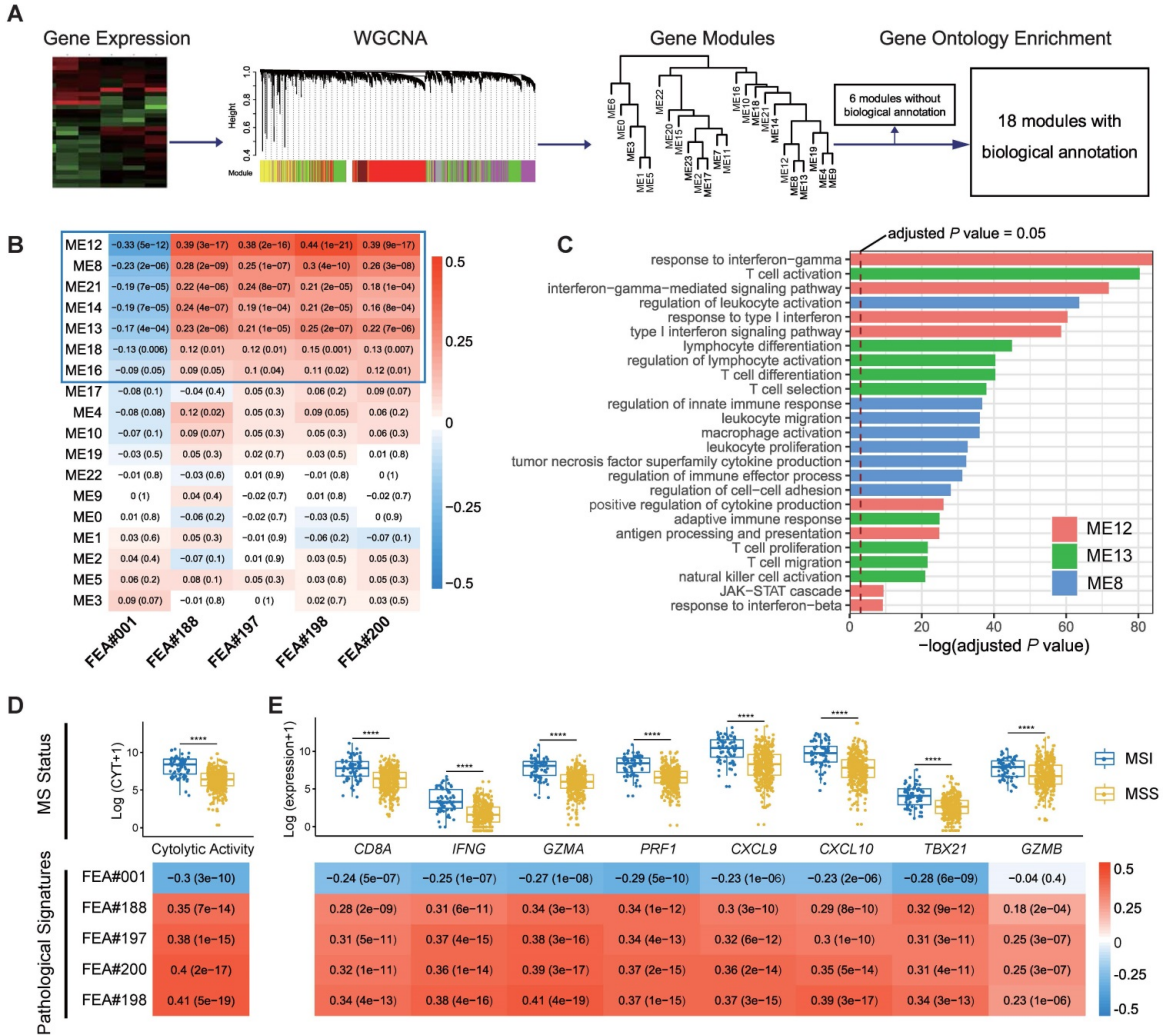
** Correlation of top pathological signatures with WGCNA-identified modules and anti-tumor immunity.** (**A**) Weighted gene co-expression network analysis (WGCNA) based on gene expression data identified gene modules with highly synergistic changes. The biological functions of these modules were annotated using Gene Ontology (GO) analyses. (**B**) Heat map of correlation coefficients (corresponding *P* values in brackets) for each pair of annotated modules and top pathological signatures. (**C**) Significantly-enriched GO terms of ME8, ME12 and ME13. The dotted line indicates the level with an adjusted *P* value of 0.05. Correlation of cytolytic activity (CYT) (**D**) and CD8^+^ T-effector genes (**E**) with MS status and top pathological signatures. The heat maps show Spearman's rank correlation coefficients, where a transition from red to blue represents positive to negative correlations. Significance values in boxplots: **** *P* < 0.0001.

We applied weighted gene co-expression network analysis (WGCNA) and identified 24 modules (Figure 5A). Gene ontology (GO) enrichment analyses were performed to annotate the modules (Table S3), among which 18 modules with biological function are retained for further analyses (Figure 5A).

Spearman's rank correlation between the 18 annotated WGCNA modules and the top five pathological signatures showed that 7 out of 18 modules are of significance, including ME12, ME8, ME21, ME14, ME13, ME18, and ME16, which were positively correlated to FEA#188/197/198/200, but negatively correlated to FEA#001 (Figure 5B). By referring to the significantly enriched GO terms of the correlated modules, we found that those molecules enriched in ME13 and ME8 were mainly related to the biological processes of immune activation, such as T cell activation and regulation of leukocyte activation (Figure 5C). As for ME12, some biological processes related to the signaling of inflammatory cytokines were significantly enriched, where the most notable was the interferon-gamma (IFN-γ) mediated pathway, namely the core IFN-γ-JAK-STAT1 signaling, which might contribute to the combination function of increased antigen processing and presentation (Figure 5C). Representative GO terms enriched in other correlated modules are shown in Figure S5.

Further investigation into the transcriptomic association of pathological signatures was conducted from the perspective of anti-tumor immunity. A strong correlation of pathological signatures with cytolytic activity (CYT) was demonstrated, which was in line with the result observed between CYT and MS status (Figure 5D). Moreover, a high degree of relevance also existed between the pathological signatures and CD8^+^ T-effector genes, consistent with the finding regarding MS status (Figure 5E). Collectively, these results indicate that the pathological signatures of the model could, to some extent, reflect the anti-tumor activity of MSI, which potentiates the efficacy of immune checkpoint inhibitors 29.

## Discussion

MSI testing can provide important information for clinical decision-making in a variety of cancers. However, the requirement of additional genetic or immunohistochemical tests limits its access to the general population. In this study, we developed a pathomics-based deep learning model which we term Ensemble Patch Likelihood Aggregation (EPLA) to predict MS status of CRC directly from histopathology images that are ubiquitously available in clinical practice, making it possible for every patient with a pathological diagnosis to receive an MSI evaluation. Furthermore, we proposed the use of transfer learning for model fine-tuning in a different population, improving its generalizability. We also explored the model interpretability from the perspective of genome and transcriptome association, giving a molecular biological explanation of our model.

In the development of a state-of-the-art method, Kather *et al.* proposed a deep-learning based majority voting method to predict MSI from histology under the assumption that all patches contribute equally to the prediction of MS status. Such assumptions might not be valid and could limit the prediction accuracy. In practice, although hundreds of patches are tiled from each WSI, most of them do not contribute much to the final prediction. In contrast, only a few key patches make the majority contribution. Our model based on multiple instance deep learning has the ability to automatically adjust the contribution of each patch to the overall WSI-level prediction in a learnable way by giving key patches higher weights, resulting in higher performances over the DL-based MV method in terms of AUC, sensitivity, and specificity. The superiority of multiple instance deep learning over DL-based MV method was also confirmed in a cohort of stomach adenocarcinoma collected from TCGA, implying the feasibility of EPLA in predicting microsatellite status across tumor types (Figure S6). Moreover, the influence of the magnification on the performance of the EPLA model was analyzed in the TCGA-COAD cohort. Notably, there was a performance degradation of our model using 5× magnification or 10× magnification, indicating that WSIs at 20× magnification better preserved the information of the microenvironment in tumors (Table S4). Therefore, we recommend this model being applied on WSIs at 20× magnification, which is also the commonly used magnification at clinical practice.

In clinical practice, different data sets could be vastly different due to the disparities between patient populations and data acquisition processes, resulting in a large performance gap for AI algorithms 38. For example, in Kather's study, an MSI classifier trained on TCGA, which was mainly made up of Western populations, and only achieved an AUC less than 0.70 in the KCCH cohort, a Japanese cohort 20. Furthermore, the histology slides in TCGA-COAD were flash-frozen slides that utilize water crystallization during the freezing process, often resulting in an altered appearance of the tissue structure as compared to the FFPE slides used in Asian-CRC which provided more tissue structure clarity. This data difference could not be effectively eliminated by only color normalization (data not shown), indicating that more advanced techniques, such as transfer learning, are necessary. As expected, EPLA showed performance degradation in Asian-CRC by simply applying the model trained on TCGA-COAD, but the results improved significantly after transfer learning. It is of clinical significance that using only a minority of the new domain data for model fine-tuning can already improve the AUC to a satisfactory level and further improvement can be expected if even more data are included, which proves that our model can be easily generalized to the complicated clinical environment, regardless of race, preparation techniques, and data acquisition techniques.

Deep learning models are often criticized for their poor interpretability, especially in mission-critical applications, such as healthcare 38. Only those models with certain interpretability can be understood, verified, and trusted by clinicians in clinical practice 21. To solve this problem, the pathological signatures, defining stable or unstable of a cancer specimen, were built during the training of the MIL model. We visualized the patches corresponding to these signatures and connected them into contours, which in turn guided us to discover the morphological features that are critical for MS status. In this way, the correlation between morphological features and the predicted MS status was investigated, through which we found that EPLA captured the information of poor differentiation in MSI tumors, in accordance with the previous finding 39. More importantly, we proposed a comprehensive molecular-level analysis including genomic and transcriptomic association analysis with pathological signatures found by AI for clinical interpretation, which could also be easily applied on other gene mutation prediction tasks. In terms of our task, the genomic association between DNA repair pathways and MSI cancers, which is exquisitely sensitive to ICB, has been verified previously 40. Moreover, MSI and its resultant TMB have been reported to underlie the response to PD-1 blockade immunotherapy 41, 42, and the INDEL mutation load is particularly associated with the extent of the response 29. Inspired by these discoveries, we confirmed the strong correlation between the pathological signatures identified by the model and these genomic biomarkers of MSI (Figure 4C and Figure S7). Despite advances in understanding of MSI at the genomic level, the process and mechanisms at the transcriptomic level remain relatively understudied. Researches on the anti-tumor effects of MSI have suggested an increased activation and infiltration of immune cells, together with an enhanced cytolytic activity as well as an up-regulation of CD8^+^ T-effector genes 29, 43. Remarkably, by analyzing the WGCNA-identified modules, the pathological signatures were demonstrated with high relevance to the expression level of IFN-γ-JAK-STAT1 signaling pathway, whose pivotal role in immune activation and response to immunotherapy has been supported by extensive evidence 44. Although IFN-γ at the same time induces feedback of up-regulation of PD-L1 on both tumor and immune cells, anti-PD-L1 therapy pertinently blocks the suppressive mechanisms, and thus inclines the balance of immune microenvironment to the inflamed phenotype 45. Furthermore, we provided evidence of a tight connection of the pathological signatures with anti-tumor activity from the perspective of transcriptomic profiles, consistent with the relationship between MS status and immunity.

The nature of AI models has limitations in our model. The performance of deep learning models largely depends on the size and quality of the training set. We still need to expand the training data to improve the accuracy and generalizability of the model. Although the model has been verified in TCGA and an Asian cohort respectively, a large prospective clinical trial is necessary before we can deploy it as a routine MSI testing method in clinical practice.

## Conclusions

In this study, we developed a pathomics-based model for MSI prediction directly from pathological images without the need for genetic or immunohistochemical tests. Using these images allows the evaluation of MS status in many more patients than was previously possible. Through the model, we identified five pathohistological imaging signatures to predict the MS status. The reliability of the model was verified in two independent cohorts and the interpretability of the model was illustrated by exploring the correlation between the pathological signatures and multi-omics characterizations. Ongoing work is attempting to further validate our model in large-cohort, prospective clinical trials.

## Supplementary Material

Supplementary figures and tables.

Supplementary table S3.

## Abbreviations

MSI: microsatellite instability
MSS: microsatellite stability
MS: microsatellite
CRC: colorectal cancer
MIL: multiple instance learning
EPLA: ensembled patch likelihood aggregation
MMR: mismatch repair
WSI: whole slide image
AI: artificial intelligence
ROI: regions of carcinoma
BCE: binary cross-entropy
PALHI: patch likelihood histogram
BoW: bag of words
NB: naïve bayes
TMB: tumor mutation burden
GO: gene ontology
CYT: cytolytic activity
DL-based MV: deep-learning based majority voting
ROC: receiver operating characteristic
AUC: area under the curve
DDR: DNA damage response and repair
HRD: homologous recombination deficiency
FEA: feature
WGCNA: weighted gene co-expression network analysis
