# Supplementary material for: Development and interpretation of a pathomics-based model for the prediction of microsatellite instability in Colorectal Cancer
Source: Theranostics. 2020 Sep 2;10(24):11080–91. doi: 10.7150/thno.49864 (PMC7532670; doi:10.7150/thno.49864)
Supplement: Supplementary file 1 — Supplementary figures and tables. [file thnov10p11080s1.pdf]

Supplementary materials for

Development and interpretation of a pathomics-based model for the prediction of microsatellite instability in colorectal cancer

Rui Cao[#], Fan Yang[#], Si-Cong Ma[#], Li Liu[#], Yu Zhao[#], Yan Li[#], De-Hua Wu, Tongxin Wang, Wei-Jia Lu, Wei-Jing Cai, Hong-Bo Zhu, Xue-Jun Guo, Yu-Wen Lu, Jun-Jie Kuang, Wen-Jing Huan, Wei-Min Tang, Kun Huang, Junzhou Huang, Jianhua Yao*, Zhong-Yi Dong*

[#] These authors contributed equally to this study

*** Corresponding authors:**

Zhong-Yi Dong (dongzy1317@foxmail.com) Department of Radiation Oncology, Nanfang Hospital, Southern Medical University, 1838 North Guangzhou Avenue, Guangzhou, 510515, China

Jianhua Yao (jianhua_yao@yahoo.com) AI Lab, Tencent, Building 12A, Shengtaiyuan, Nanshan District, Shenzhen, 518057, China

Table of Contents

Figure S1. Scale independence and mean connectivity of the WGCNA network for soft threshold determination. 3

Figure S2. Adjacency heatmap of the WGCNA identified modules... 4

Figure S3. Heat maps of representative discrepant cases between EPLA and DL-based MV........ 5

Figure S4. Prediction performance with regards to tumor stage in the TCGA-COAD cohort and the Asian-CRC cohort. 6

Figure S5. Representative enriched Gene Ontology (GO) terms in other correlated modules (ME14, ME16, ME18, and ME21). 7

Figure S6. Prediction performance of EPLA in stomach adenocarcinoma. 8

Figure S7. Correlation between the pathological signatures and tumor mutation burden (TMB). 9

Table S1. Summary of the TCGA-COAD and Asian-CRC cohorts. 10

Table S2. Sensitivities and specificities of different models with optimal cut-offs evaluated in the TCGA-COAD test set. 11

Table S3. Gene ontology (GO) terms enriched in the WGCNA-identified modules. 12

Table S4. Summary of the EPLA using different magnifications in the TCGA-COAD test set. . 13

Figure S1. Scale independence and mean connectivity of the WGCNA network for soft threshold determination.

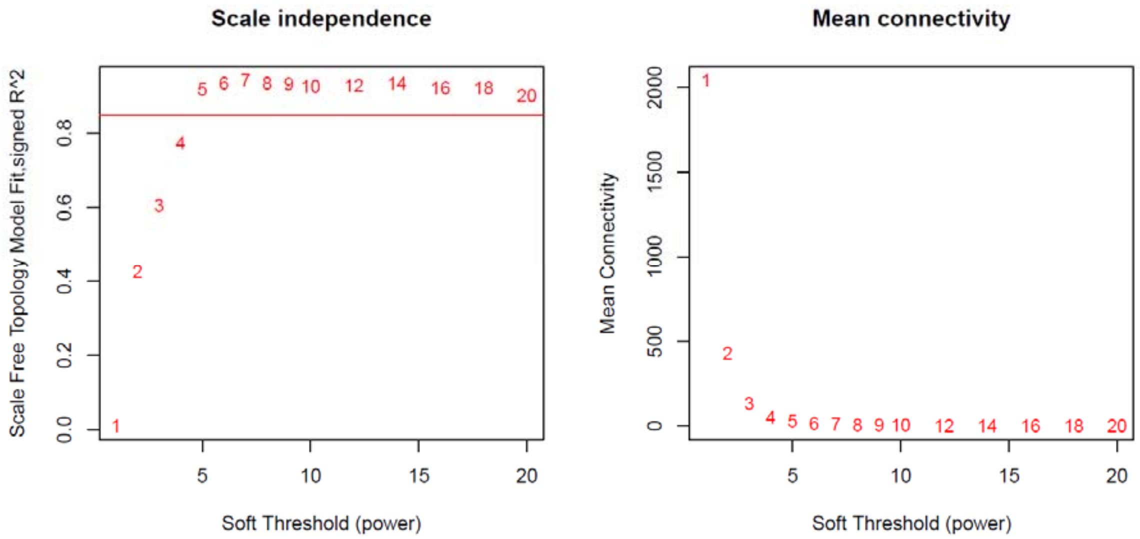

Figure S2. Adjacency heatmap of the WGCNA identified modules. WGCNA: gene co-expression network analysis; ME: module.

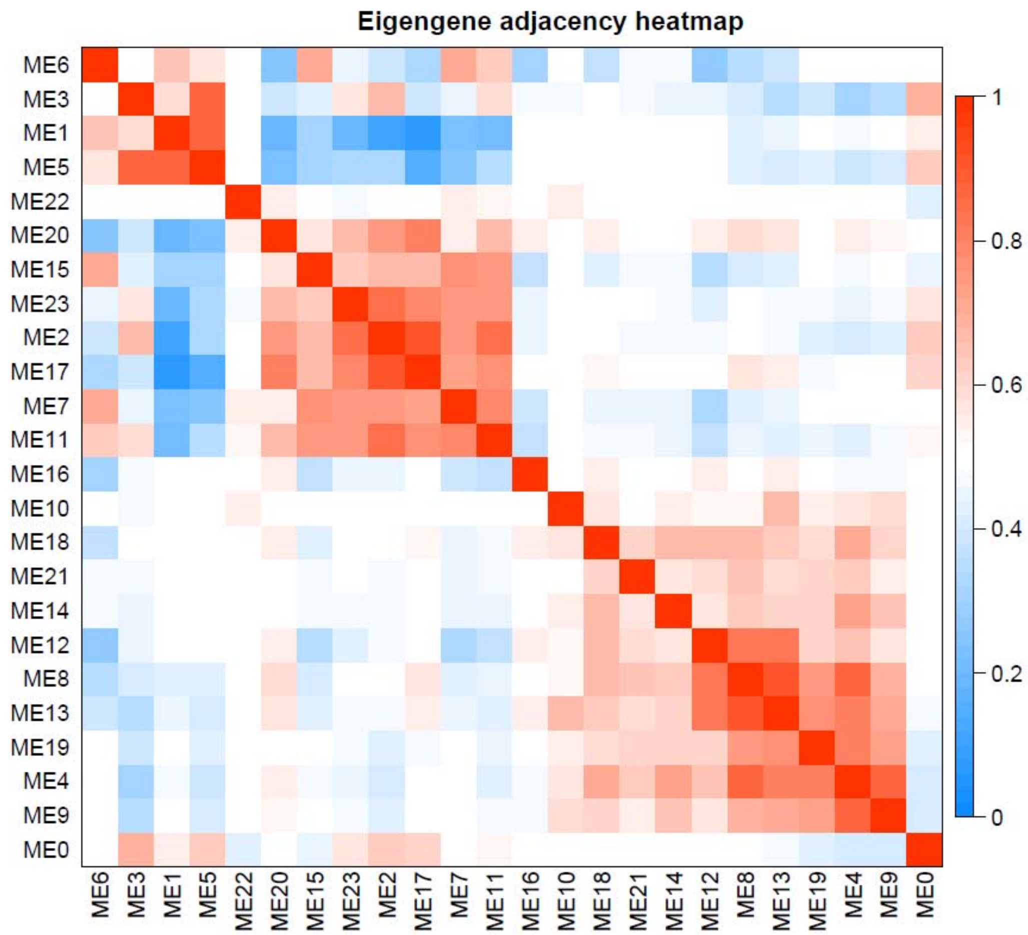

Figure S3. Heat maps of representative discrepant cases between EPLA and DL-based MV. EPLA: Ensemble Patch Likelihood Aggregation; DL-based MV: Deep-Learning based Majority Voting.

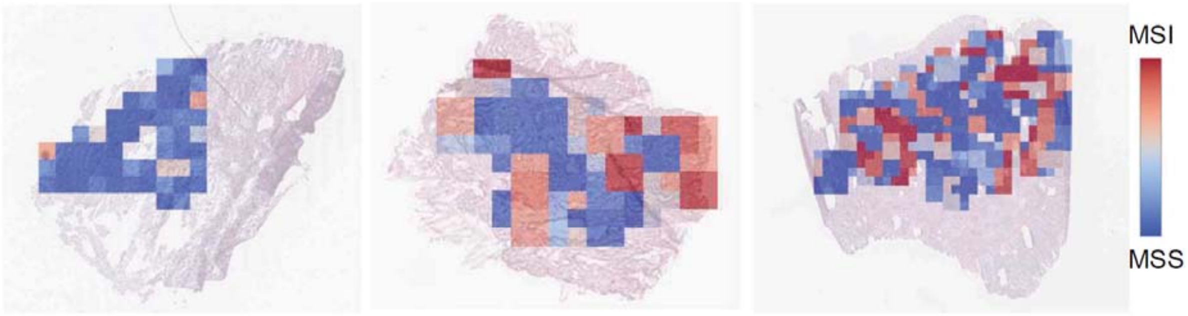

MSIsensor score: 32.47 (MSI)	MSIsensor score: 21.62 (MSI)	MSIsensor score: 0.35 (MSS)
DL-based MV = 0.0926 (MSS)	DL-based MV = 0.3330 (MSS)	DL-based MV = 0.5063 (MSI)
EPLA = 0.6097 (MSI)	EPLA = 0.6338 (MSI)	EPLA = 0.2637 (MSS)

Figure S4. Prediction performance with regards to tumor stage in the TCGA-COAD cohort and the Asian-CRC cohort. Area under the receiver operating curve (AUC) of EPLA in (A) the stage I-III cases from the TCGA-COAD cohort, (B) the stage IV cases from the TCGA-COAD cohort, (C) the stage I-III cases from the Asian-CRC cohort, and (D) the stage IV cases from the Asian-CRC cohort. TCGA: The Cancer Genome Atlas; EPLA: Ensemble Patch Likelihood Aggregation; COAD: colon adenocarcinoma; CRC: colorectal cancer.

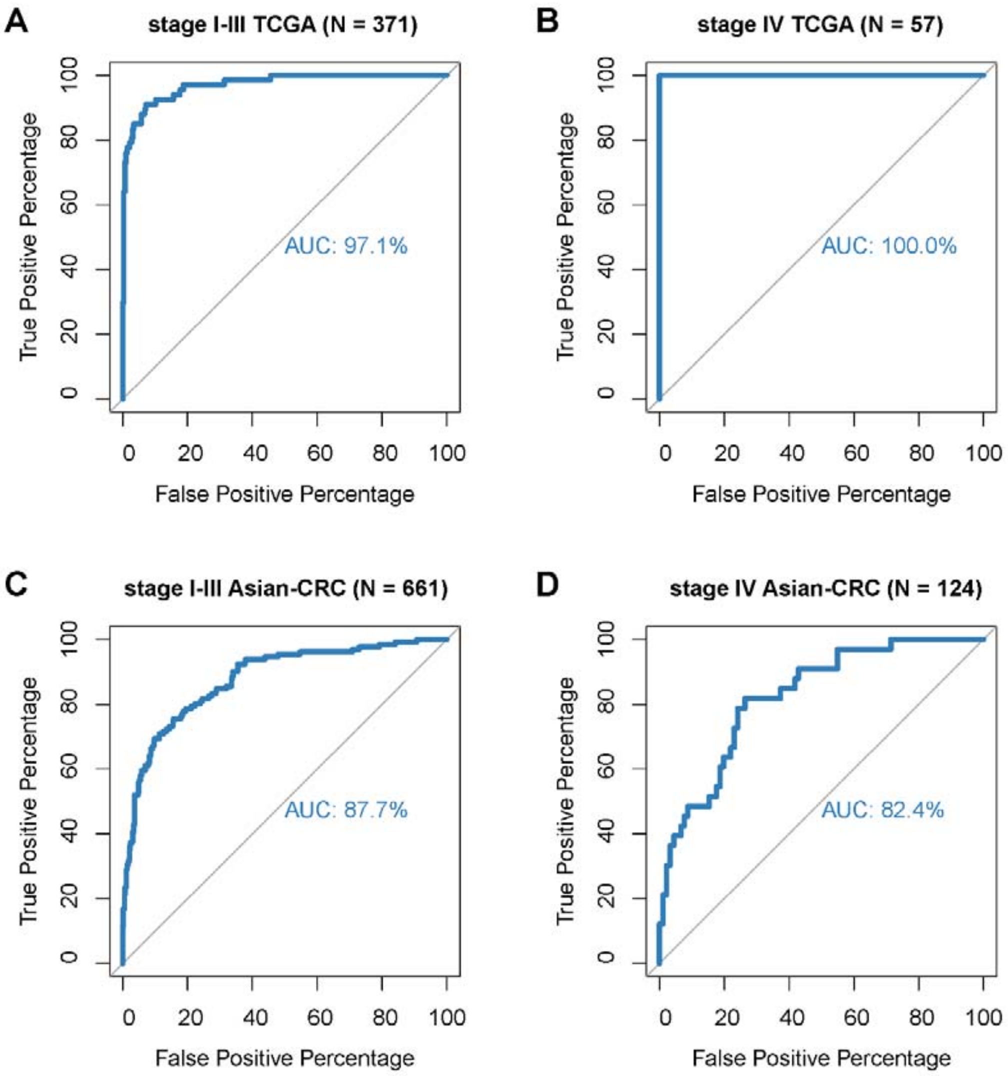

Figure S5. Representative enriched Gene Ontology (GO) terms in other correlated modules (ME14, ME16, ME18, and ME21). The Benjamini-Hochberg method was used to adjust *P* value for controlling false discover rate. ME: module.

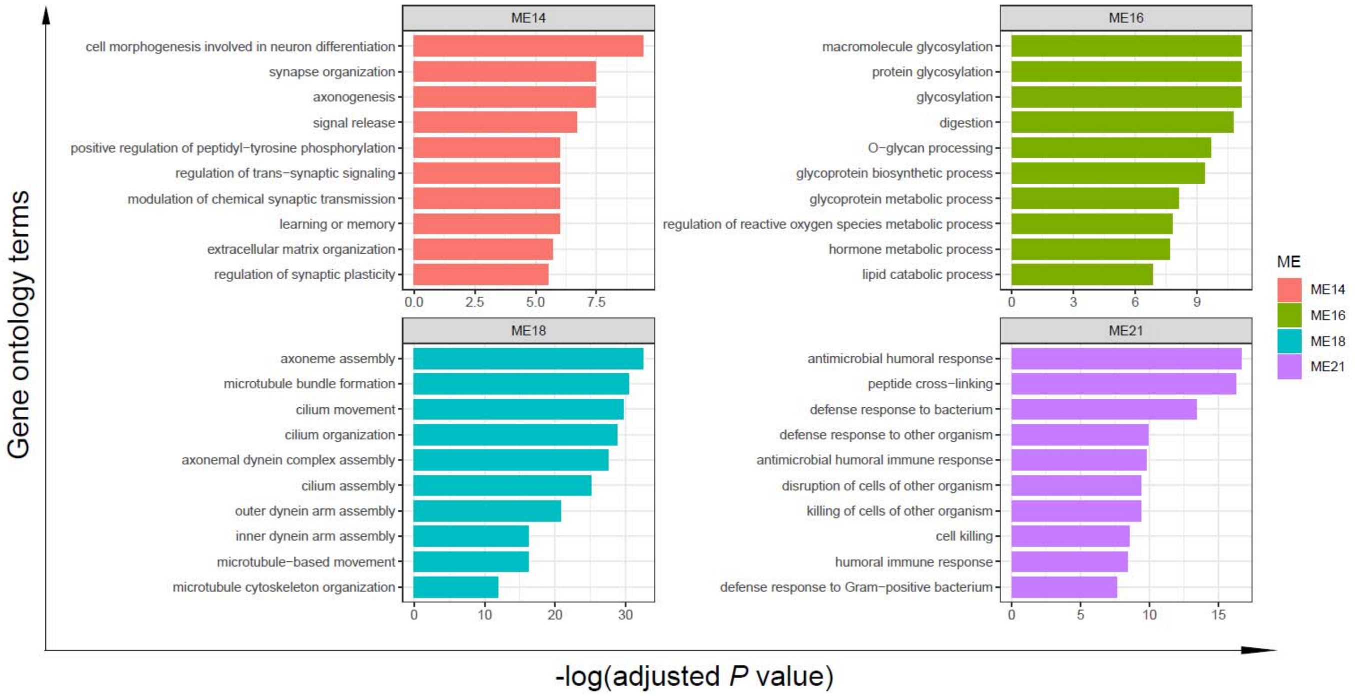

Figure S6. Prediction performance of EPLA in stomach adenocarcinoma. (A) Receiver operating characteristic (ROC) curve of EPLA in the TCGA-STAD test set. (B) Comparison of the performance of EPLA with the state-of-the-art DL-based MV method. TCGA: The Cancer Genome Atlas; STAD: stomach adenocarcinoma; AUC: area under curve; CI: confidence interval; EPLA: Ensemble Patch Likelihood Aggregation; DL-based MV: Deep-Learning based Majority Voting.

A

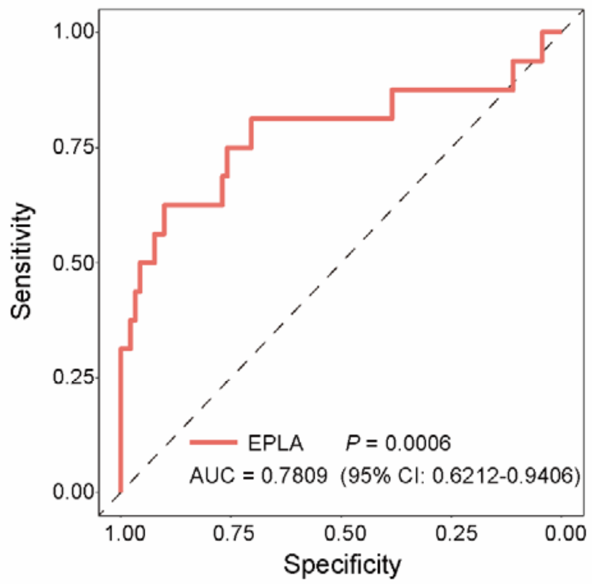

B

Method	Cohort Name	Size (MSS:MSI)	ROCAUC	95% CI
EPLA	TCGA-STAD	355 (302:53)	0.7809	0.6212-0.9406
DL-based MV	TCGA-STAD	355 (302:53)	0.7486	0.5787-0.9185

Figure S7. Correlation between the pathological signatures and tumor mutation burden (TMB). Boxplots showing the distribution of the top five pathological signatures, extracted by the model, stratified by TMB with a threshold of 30 mut/Mb.

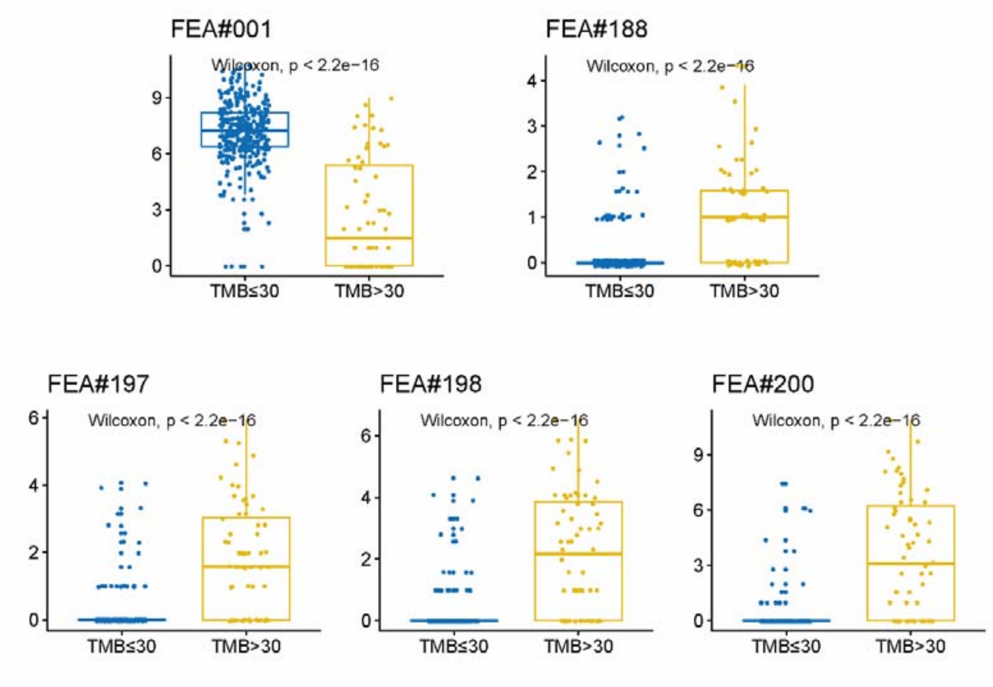

Table S1. Summary of the TCGA-COAD and Asian-CRC cohorts.

Cohort	Material	Annotated WSI	MSS	MSI	Patches
					min, 25%, 50%, 75%, max
TCGA-COAD	Frozen slides	429	358	71	22, 143, 229, 398, 2357
Asian-CRC	FFPE	785	621	164	5, 179, 338, 608, 3718

Abbreviations: FFPE: formalin-fixed paraffin-embedded; WSI: whole slide image; MSI: microsatellite instability; MSS: microsatellite stability.

Table S2. Sensitivities and specificities of different models with optimal cut-offs evaluated in the TCGA-COAD test set.

	**DL-based MV**	**PALHI pipeline**	**BoW pipeline**	**EPLA**
Sensitivity	0.82	0.86	0.73	0.91
Specificity	0.75	0.76	0.9	0.77

Abbreviations: DL-based MV: deep-learning based majority voting; PALHI: PAtch Likelihood Histogram; Bag of Words (BoW); EPLA: Ensembled Patch Likelihood Aggregation.

Table S3. Gene ontology (GO) terms enriched in the WGCNA-identified modules. The Benjamini-Hochberg method was used to adjust *P* value for controlling false discover rate, and those GO terms with adjusted *P* values lower than 0.05 were considered significantly enriched in a particular module. WGCNA: gene co-expression network analysis.

(Table S3 is provided in a separate Microsoft Excel file because of its large size.)

Table S4. Summary of the EPLA using different magnifications in the TCGA-COAD test set.

Method	Cohort Name	Magnification	ROCAUC	95% CI
EPLA	TCGA-COAD	20×	0.8848	0.8185-0.9512
EPLA	TCGA-COAD	10×	0.7710	0.6646-0.8774
EPLA	TCGA-COAD	5×	0.6801	0.5544-0.8058

Abbreviations: EPLA: Ensembled Patch Likelihood Aggregation; ROC: receiver operating characteristic; AUC: area under curve; CI: confidence interval.
